# Recurrent Intramuscular Hematoma Revealing Moderate Congenital Factor XIII Deficiency

**DOI:** 10.7759/cureus.95841

**Published:** 2025-10-31

**Authors:** Nada Ben Halima, Salma Riahi, Ines Mazlout, Imen Mgarrech, Amina Bouatay

**Affiliations:** 1 Laboratory of Hematology and Blood Bank/Laboratory of Parasitology, Sahloul Hospital, Faculty of Medicine of Sousse, University of Sousse, Sousse, TUN; 2 Laboratory of Hematology and Blood Bank, Sahloul Hospital, Faculty of Medicine of Sousse, University of Sousse, Sousse, TUN; 3 Department of Cardiovascular and Thoracic Surgery, Sahloul Hospital, Faculty of Medicine of Sousse, University of Sousse, Sousse, TUN; 4 Laboratory of Hematology and Blood Bank, Sahloul Hospital, Faculty of Pharmacy of Monastir, University of Monastir, Sousse, TUN

**Keywords:** diagnosis, factor xiii, hematoma, hemorrhage, treatment

## Abstract

Severe congenital factor XIII deficiency is a rare blood coagulation disorder that poses a risk of life-threatening bleeding complications. Conversely, patients with mild or moderate factor XIII deficiency frequently exhibit delayed or provoked bleeding patterns. This case report presents a patient who received a delayed diagnosis of moderate factor XIII deficiency following significant bleeding episodes in the postoperative period. We will examine the symptoms indicative of mild and moderate factor XIII deficiency, as well as the current therapeutic options. This discussion emphasizes the vital importance of early diagnosis for achieving optimal management and improved patient outcomes.

## Introduction

Congenital coagulation factor XIII (FXIII) deficiency is a rare autosomal recessive disorder with a global prevalence of approximately two per million inhabitants [1]. The most indicative symptoms include umbilical hemorrhage at cord separation and intracranial hemorrhage [2,3]. In addition to the hemorrhagic manifestations, this condition is associated with wound-healing disorders and, in pregnant women, recurrent miscarriages [4]. Congenital factor XIII deficiency is categorized into three clinical forms. Severe deficiency, defined by undetectable FXIII activity (<1%), is typically associated with spontaneous major bleeding. Moderate deficiency, characterized by FXIII activity levels below 30%, may result in either spontaneous or induced bleeding. In contrast, minor deficiency, with FXIII activity above 30%, is most often asymptomatic [5].

Recently, minor factor XIII deficiency (FXIII activity between 30% and 60%) has garnered considerable attention, as it is clinically and biologically distinct from severe congenital factor XIII deficiency. This form has been largely overlooked due to the mild and non-specific nature of its symptoms. Patients with minor deficiencies do not experience spontaneous bleeding; however, they may face delayed bleeding after surgical procedures or traumatic events. Moreover, in women, minor factor XIII deficiency may play a role in failures during in vitro fertilization. Menorrhagia is a common symptom, particularly among patients with other underlying hemostatic disorders [6].

Beyond its clinical presentation, FXIII deficiency also poses important public health and diagnostic challenges, particularly in low-income regions where limited access to specialized assays and awareness among clinicians often leads to underdiagnosis or delayed recognition of mild and moderate forms. Addressing these constraints is essential to improving patient outcomes and guiding appropriate management strategies.

In this observation, we underscore the seriousness of this condition when it is accompanied by additional bleeding risk factors, such as anticoagulant therapy and postoperative or post-traumatic circumstances. We also highlight the critical importance of timely diagnosis to ensure optimal management.

## Case presentation

The patient was a 64-year-old male from southern Tunisia, born to a first-degree consanguineous marriage, with no known family history of hemorrhagic conditions. His medical history included atrial fibrillation (AF) managed with anti-vitamin K (AVK) therapy and a recurrent post-traumatic hematoma of the right thigh, with the first episode occurring four weeks after the trauma and a second recurrence three weeks later within the same year. Upon admission, the patient was observed to have tachycardia, with a heart rate of 100 beats per minute and blood pressure measuring 100/60 mmHg. He demonstrated preserved diuresis and reported no chest pain or episodes of syncope. There were no signs of right or left heart failure. The patient was eupneic, exhibiting a respiratory rate of 12 breaths per minute, with clear vesicular breath sounds noted on auscultation. Pulses in both lower limbs were present and symmetrical. Examination of the limbs revealed swelling in the right thigh, which was firm to the touch and accompanied by a purplish ecchymosis measuring approximately 20 cm in diameter, without any signs of inflammation on the skin. The patient remained conscious and well-oriented throughout the examination, which was otherwise unremarkable. The patient was initially managed surgically by orthopedic surgery for the drainage of the hematoma. However, following the failure of this treatment, evidenced by two recurrences, the patient was referred to the department of cardiovascular and thoracic surgery for a thorough investigation of a potential vascular anomaly. The patient’s vitamin K antagonist (AVK) was interrupted, and therapeutic low-molecular-weight heparin (LMWH) was initiated as a perioperative “bridging” agent to allow rapid reversal of anticoagulation prior to the planned operation. LMWH was maintained at a curative dose due to the underlying AF. During the initial hemostatic assessment, the patient's hemoglobin level was 10.4 g/dL, with a mean corpuscular volume (MCV) of 89 fL. The white blood cell count (WBC) was measured at 6,000/mm³, and the platelet count was 121 G/L. The prothrombin time (PT) was recorded at 76%, while the activated partial thromboplastin time (aPTT) was 26.3 seconds, resulting in a patient/control (P/C) ratio of 0.9. The platelet occlusion test was normal, and the coagulation factors of the prothrombin complex (factor II, V, VII, and X), alongside the fibrinogen assays, also yielded normal findings. Von Willebrand factor (VWF) ristocetin cofactor activity assay (VWF Rco) was within normal limits. However, upon performing a factor XIII antigen assay using an automated immunological method on ACL TOP 750 (Werfen, Barcelona, Spain), a factor XIII level of 26.2% was revealed, with results consistent across two different samples. A mixed correction test (patient factor XIII at 26.2%, control factor XIII at 67%, and mixed factor XIII at 52.8%) suggested the presence of a congenital factor XIII deficiency (Table 1). The remainder of the laboratory evaluations showed no abnormalities, and surgical exploration excluded any vascular causes for the bleeding. A protocol involving the transfusion of fresh frozen plasma (FFP) was implemented; however, due to the severity of the hematoma and the unsuccessful surgical intervention, a mid-thigh amputation was ultimately chosen. Initially, the patient showed positive progress, with bleeding ceasing and healing commencing. Unfortunately, the patient succumbed to severe sepsis complicated by septic shock.

**Table 1:** Laboratory findings of the patient with corresponding reference ranges (according to our laboratory standards). P/C = patient/control ratio. Reference ranges are based on the standards of our institution's laboratory.

Parameter	Result	Reference range	Interpretation
Hemoglobin (Hb)	10.4 g/dL	13–17 g/dL	Anemia, worsened by recurrent bleeding
Mean corpuscular volume (MCV)	89 fL	80–100 fL	Normocytic
White blood cells (WBC)	6000/mm³	4000–10,000/mm³	Normal
Platelets	121 G/L	150–400 G/L	Mild thrombocytopenia
Prothrombin time (PT)	76%	70–100%	Normal
Activated partial thromboplastin time (aPTT)	26.3 sec (P/C = 0.9)	P/C = 0.8-1.2	Normal
Factor II	80%	79-131%	Normal
Factor V	95.1%	62-139%	Normal
Factor VII	52.6%	50-129%	Normal
Factor X	85.3%	77-131%	Normal
Fibrinogen	2.15	2–4 g/L	Normal
Von Willebrand factor activity: ristocetin cofactor activity (vWF:RCo)	88%	50–150%	Normal
Von Willebrand factor antigen (vWF Ag)	110%	50–150%	Normal
Factor XIII	26.2%	70–140%	Decreased

## Discussion

Factor XIII, also referred to as fibrin-stabilizing factor, is an essential component of the blood coagulation process [7]. It functions by stabilizing the fibrin clot formed after the coagulation cascade through several mechanisms: it facilitates the formation of covalent bonds between the α and γ monomers of fibrin, catalyzes the binding of α2-antiplasmin to fibrin to inhibit fibrinolysis, and allows the fibrin clot to anchor to subendothelial proteins such as fibronectin, vitronectin, and collagen [8]. Consequently, FXIII plays a significant role in wound healing and tissue repair [9]. Congenital FXIII deficiency is a rare autosomal recessive disorder that can manifest in various clinical forms. In 80% of severe cases, symptoms typically present as hemorrhage during the neonatal period, specifically when the umbilical cord detaches, or through spontaneous intracranial hemorrhage in 30% of cases [1]. Less severe or moderate forms may present with recurrent mucosal bleeding, ecchymosis, hemarthrosis, muscle hematomas, and, characteristically, delayed bleeding occurring 12 to 36 hours after trauma or surgery [10].

Diagnosing FXIII deficiency can be challenging without a strong clinical suspicion, and many cases remain undiagnosed, potentially resulting in the late onset of life-threatening complications [11]. Heterozygous FXIII deficiency, while generally less severe than the congenital form, can still lead to serious delayed bleeding, particularly when accompanied by other bleeding risk factors. According to data from the international Rare Bleeding Disorders Database (RBDD), bleeding complications have only been reported in patients with FXIII levels averaging 16.8% (range = 0-37.1%), typically associated with post-traumatic events or linked to anticoagulant or antiplatelet therapy [5].

In our case, the diagnosis was ultimately made late, following the recurrence of a muscle hematoma in the right thigh, which occurred after minor trauma and coincided with the patient being started on anticoagulants (AVK) for atrial fibrillation. Additionally, there was no notable history of prolonged umbilical cord bleeding, bruising, hematomas, or mucosal bleeding during childhood. Aside from consanguinity, the family history did not indicate any signs of hemorrhagic syndrome in the siblings. The patient's geographical background (southern Tunisia) aligns with existing literature, as this region shows a particularly high prevalence of FXIII deficiency [12].

Unlike other congenital coagulation factor deficiencies, FXIII deficiency is not identified through standard hemostasis tests such as PT, aPTT, and fibrinogen levels, all of which typically remain normal since FXIII functions after the initial clot formation [3]. Diagnosis relies on a specific assessment of FXIII activity, informed by a suggestive medical history and clinical signs, along with a normal coagulation profile. It is advisable to investigate potential genetic abnormalities using molecular biology techniques to confirm the diagnosis, although this approach can be costly and may not be accessible in all laboratories. Currently, prenatal screening is not conducted due to the rarity of the disease, the accreditation required to perform such screenings, and the unclear hemorrhagic risk associated with the procedure [8]. Current recommendations highlight that prophylactic therapy should be strongly considered in patients with activity <5% because of the high risk of intracranial hemorrhage, but also in selected patients with levels up to 15% if they have a history of major bleeding [13]. There is limited literature on the long-term prognosis for patients with heterozygous FXIII deficiency, but it appears to be primarily associated with hemorrhagic complications in the absence of proactive long-term treatment. For instance, in a retrospective ICU-based cohort of “mild acquired FXIII deficiency” (activity levels <70%) patients, even modest reductions in FXIII activity were associated with increased transfusion needs and bleeding risks [14].

The primary treatment for congenital FXIII deficiency is replacement therapy using FXIII concentrate. The in vivo half-life of FXIII ranges from 11 to 14 days, allowing for relatively infrequent administration of these concentrates. There are two available types: a plasma-derived concentrate (pFXIII), known as Fibrogammin®, and a recombinant concentrate (rFXIII), either Catridecacog® or Novothirteen®. In cases of acute hemorrhage, the recommended initial dose is 20 to 40 IU/kg for pFXIII and 35 IU/kg for rFXIII [8]. However, access to this treatment can be challenging in low- and middle-income countries due to high costs and limited availability. In emergencies, treatment with FFP at a dose of 20 mL/kg may serve as an alternative [8].

In the case of our patient, the unavailability of FXIII concentrate led us to use FFP transfusions. Unfortunately, the delayed diagnosis and the failure to administer the specific treatment promptly resulted in severe bleeding episodes from recurrent muscle hematomas, which ultimately necessitated amputation. There is limited literature on the long-term prognosis for patients with heterozygous FXIII deficiency, but it appears to be primarily associated with hemorrhagic complications in the absence of proactive long-term treatment [15].

## Conclusions

FXIII deficiency continues to present challenges in both diagnosis and treatment. Although it is extremely rare, the presence of recurrent hematomas or spontaneous or provoked bleeding should raise suspicions about the diagnosis, especially when standard coagulation tests yield normal results. A rapid, sensitive, and standardized FXIII assay would facilitate the swift initiation of targeted treatment with FXIII concentrates. If these are unavailable, using FFP can help mitigate the risk of severe and potentially life-threatening hemorrhagic complications. FXIII deficiency should be suspected in any patient presenting with recurrent bleeding despite normal PT/aPTT, particularly in the presence of consanguinity or anticoagulation therapy.
